# Evaluating the impact of DREAMS on HIV incidence among young women who sell sex: protocol for a non-randomised study in Zimbabwe

**DOI:** 10.1186/s12889-018-5085-6

**Published:** 2018-01-31

**Authors:** Bernadette Hensen, James R. Hargreaves, Tarisai Chiyaka, Sungai Chabata, Phillis Mushati, Sian Floyd, Isolde Birdthistle, Joanna Busza, Frances Cowan

**Affiliations:** 1 Department of Clinical Research, Infectious and Tropical Disease, London School of Hygiene and Tropical Medicine, Keppel Street, London, WC1E 7HT UK; 2 Department of Social and Environmental Health Research, Public Health and Policy, London School of Hygiene and Tropical Medicine, London, UK; 3 The Centre for Sexual Health & HIV/AIDS Research (CeSHHAR) Zimbabwe, Harare, Zimbabwe; 4 Department of Infectious Disease Epidemiology, Epidemiology and Population Health, London School of Hygiene and Tropical Medicine, London, UK; 5 Department of Population Health, Epidemiology and Population Health, London School of Hygiene and Tropical Medicine, London, UK; 6 Department of International Public Health, Liverpool School of Tropical Medicine, Liverpool, UK

**Keywords:** HIV prevention, Young women who sell sex, Zimbabwe, Complex intervention

## Abstract

**Background:**

“Determined, Resilient, AIDS-free, Mentored and Safe” (DREAMS) is a package of biomedical, social and economic interventions offered to adolescent girls and young women aged 10–24 years with the aim of reducing HIV incidence. In four of the six DREAMS districts in Zimbabwe, DREAMS includes an offer of oral pre-exposure prophylaxis (DREAMS+PrEP), alongside interventions to support demand and adherence, to women aged 18–24 who are at highest risk of HIV infection, including young women who sell sex (YWSS). This evaluation study addresses the question: does the delivery of DREAMS+PrEP through various providers reduce HIV incidence among YWSS Zimbabwe? We describe our approach to designing a rigorous study to assess whether DREAMS+PrEP had an impact on HIV incidence.

**Methods:**

The study design needed to account for the fact that: 1) DREAMS+PrEP was non-randomly allocated; 2) there is no sampling frame for the target population for the evaluation; 3) there are a small number of DREAMS districts (*N* = 6), and 4) DREAMS+PrEP is being implemented by various providers. The study will use a cohort analysis approach to compare HIV incidence among YWSS in two DREAMS+PrEP districts to HIV incidence among YWSS in non-DREAMS comparison sites. YWSS will be referred to services and recruited into the cohort through a network-based (respondent-driven) recruitment strategy, and followed-up 12- and 24-months after enrolment. Women will be asked to complete a questionnaire and offered HIV testing. Additional complications of this study include identifying comparable populations of YWSS in the DREAMS+PrEP and non-DREAMS comparison sites, and retention of YWSS over the 24-month period. The primary outcome is HIV incidence among YWSS HIV-negative at study enrolment measured by repeat, rapid HIV testing over 24-months. Inference will be based on plausibility that DREAMS+PrEP had an impact on HIV incidence. A process evaluation will be conducted to understand intervention implementation, and document any contextual factors determining the success or failure of intervention delivery.

**Discussion:**

HIV prevention products of known efficacy are available. Innovative studies are needed to provide evidence of how to optimise product use through combination interventions to achieve population impact within different contexts. We describe the design of such a study.

## Background

In public health, one aim of implementation science studies, including impact evaluations, is to understand whether and how interventions with known efficacy should be delivered to have a population-level impact in real-life settings. Interventions supporting delivery are often complex, taking the form of a package of strategies to support demand for, use and adherence to prevention behaviours and/or technologies. Contextual adaptation for effective delivery may be critical to achieve impact. Randomised allocation may not be feasible in some settings, posing challenges to such studies. Implementation science studies therefore require innovation in study design, and pre-published protocols for these studies are particularly important yet less commonly published than for randomised trials.

In this protocol paper, we describe the evaluation of a complex and ambitious intervention package being delivered by multiple providers with the aim of reducing HIV infection rates among high-risk young women in Zimbabwe. We outline challenges faced in designing a rigorous study to estimate the impact of the intervention in this situation, and describe our proposed design, analysis strategy and approach to inference.

Across sub-Saharan Africa, adolescent girls and young women (AGYW) are at disproportionately higher risk of HIV than older females and their male peers [1, 2]. This increased risk is driven by a combination of biological, behavioural and structural factors [3]. At highest risk of infection are young female sex workers (FSW) and other young women who sell sex (YWSS) for financial or material resources [4]. Relative to older FSW, young FSW and YWSS aged under 25 years are at increased risk due to economic vulnerability, sexual partnerships with older men more likely to than younger men to have prevalent infection, and reduced skills in condom use negotiation [5–8]. Limited access to available health services for fear of stigma and discrimination further increases their risk of HIV and other health outcomes [5, 7]. In Zimbabwe, our experience is that YWSS, those under 25 years as well as those who have most recently begun sex work, may not identify as sex workers. Consequently, they are less likely to access sex work specific services [4, 9]. Younger FSW are also less frequently recruited to research studies of FSW [10, 11]. As such, YWSS are particularly vulnerable to new infection as well as being hidden and complex to conduct evaluation studies with. We anticipate that their risk of new HIV infection is very high; our estimates among young FSW suggest an incidence perhaps as high as 10% per year [9].

In 2015, WHO recommended the use of tenofovir-based oral pre-exposure prophylaxis (PrEP) for individuals at substantial risk of HIV as part of combination HIV prevention [12] based on a systematic review of PrEP studies among a range populations and settings [13]. The review found that PrEP was effective at reducing HIV risk across sexual exposures, PrEP regimen, dosing, and mode of acquisition, and that increased adherence was associated with a demonstrable increase in effectiveness [13]. In trials with adherence > 70%, PrEP reduced risk of infection by 70% (RR = 0.30, 95% CI: 0.21–0.45, *p* = 0.001) [13]. While there is no doubt that, when taken, PrEP is biologically effective in women, pharmacokinetic studies have indicated lower concentrations of tenofovir in vaginal than rectal tissues [14–16] suggesting that adherence is particularly important in women. Evidence from treatment scale-up suggests that younger people find it more difficult to adhere to treatment than older individuals [17], and may require increased adherence support tailored to their age group and lifestyle. In settings where PrEP is available, implementation research is needed to translate the efficacy of PrEP into population-level impact through increased demand and adherence among those at highest risk of HIV, including YWSS. To achieve population-level impact, combined strategies that deliver services that are acceptable and accessible to YWSS, generate demand for, and support use of and adherence to PrEP should be considered [8, 18].

The DREAMS (“Determined, Resilient, Empowered, AIDS-free Mentored, and Safe”) initiative is a package of HIV prevention interventions that aim to synergistically address social, economic, behavioural and biological risk factors that place young women at heightened risk of infection [19, 20]. DREAMS is being implemented in ten sub-Saharan African countries with DREAMS aiming to reduce HIV incidence by 40% among AGYW aged 15–24 years [20]. The package includes social protection (interventions to support young women to remain or return to school, or acquire vocational skills), clinical and prevention services for gender-based violence (GBV), and HIV prevention (HIV testing, STI screening and treatment, condoms). Various providers are implementing these combined interventions in real-world conditions, targeting the most vulnerable, high-risk AGYW. In some settings, including Zimbabwe, PrEP is a component of the package offered to AGYW aged 18 to 24 years at highest risk of HIV (DREAMS+PrEP). This paper outlines the design of a non-randomised, study to estimate the impact of DREAMS+PrEP on HIV incidence among YWSS in two sites in Zimbabwe and to compare this to incidence in sites where DREAMS is not available.

### The DREAMS+PrEP initiative in Zimbabwe

In Zimbabwe, DREAMS is being delivered in six districts by seven implementing partners (IP). IP identify young women who are eligible and likely to benefit from DREAMS, determine their needs and refer them to other IP as appropriate. The route into DREAMS varies according to the specific focus of the IP, for example, entry into DREAMS may be through schools, clinics, HIV testing services or the community more generally. Through this referral process, DREAMS interventions can be “layered” dependent on need. DREAMS targets all vulnerable AGYW aged 10–24 years. In four of the six DREAMS districts, PrEP is being offered to women at highest risk of infection aged 18–24 years, particularly YWSS. PrEP is delivered by PSI Zimbabwe. YWSS are identified through community outreach and mobilisation led by The Centre for Sexual Health and HIV/AIDS Research (CeSHHAR). Community mapping guided the community outreach to identify hotspots where sex is exchanged for material resources. Importantly mapping was not limited to sex work venues.

### Network-based recruitment for referral to services and cohort recruitment

We hypothesise that YWSS exist in sizeable numbers in the DREAMS sites and that they are inter-connected through social networks. To identify these young women, refer them to DREAMS+PrEP services, and recruit them into the impact evaluation study, we will use a network-based recruitment (RDS) strategy that uses social networks to identify and recruit eligible women. At the time of the design of this study, the DREAMS programme selected the 18–24 age cut-off for PrEP. The DREAMS target was to initiate 1400 women aged 18–24 on PrEP, of which 80% would be referred from two DREAMS+PrEP sites. In the two DREAMS+PrEP sites, PSI will deliver PrEP. To support uptake and adherence to PrEP, community mobilisation activities, PrEP adherence support groups and peer outreach services will be specifically targeted to YWSS. *Sisters with a Voice* (*Sisters*) programme, an existing national FSW programme that offers HIV care and prevention services, will deliver these services. Initiated in 2009, *Sisters* provides HIV targeted services to FSW in 36 sites across Zimbabwe in line with WHO guidance [4, 9]. The *Young Sisters* programme offers services specifically tailored for younger women aged 15–19 years [4].

**Fig. 1:**
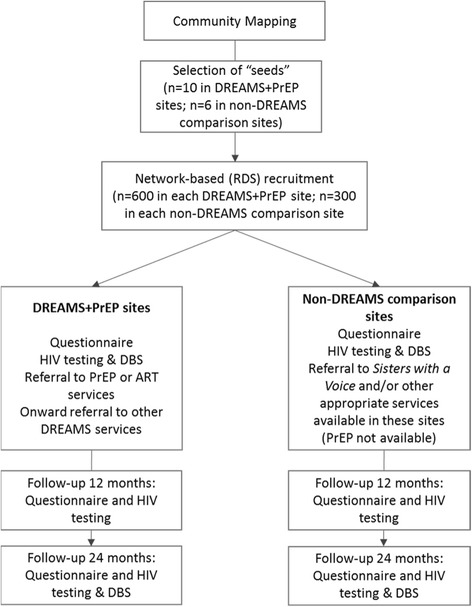
Schematic of the processes involved in this study to identify, reach and refer young women who sell sex to services and recruit these same women into an impact evaluation cohort

The community mapping allowed for the identification and recruitment of “seeds” to start the network-based recruitment process (Fig. 1). A “seed” is a participant purposively selected to start recruitment. Seeds will be drawn from different ages (between 18 and 24 years), types of selling sex/exchanging sex for resources and geographical spread across each site as identified in community mapping. Each seed will be offered two coupons and asked to recruit two young women who they think meets the inclusion criteria [21], namely: the recruit should be a young woman aged 18–24 whom they know (defined as seeing each other at least once a month) who also exchanges sex with men for money and/or resources. Our definition of “selling sex” includes young women who self-identify as FSW and extends to young women who have sex with men in exchange for money and/or resources and report the sex would not occur in the absence of this exchange (but do not think of themselves as sex workers). Women receiving a coupon who attend the study site for enrolment (“recruits”) will also be given two coupons to recruit two peers. In all impact evaluation sites, six iterations of this process (“waves”) will be completed to refer YWSS to services and recruit the sample size required for the impact evaluation as described below.

Once reached, women attending the study site will be referred to DREAMS+PrEP interventions dependent on need and recruited to the impact evaluation. The same strategy is being implemented in four non-DREAMS comparison sites, with YWSS referred to the *Sisters* programme and/or other appropriate services available in these sites. Services offered at *Sisters* clinics include: STI screening and treatment, HIV testing with referrals to government services for treatment, free condoms and contraception, and legal advice supported by a network of peer educators.

### Key considerations for study design

We seek to estimate the impact of the DREAMS+PrEP intervention on HIV incidence among YWSS aged 18–24. The DREAMS+PrEP intervention includes identifying high risk women, referral for an offer of PrEP for those testing negative, and referral to other DREAMS interventions, depending on need and availability, that will support use and adherence through a variety of behavioural and structural interventions. This impact evaluation has four main design challenges. First, it was not possible to randomise study sites to receive the DREAMS intervention. DREAMS districts were selected through country-level consultation and based on population of AGYW and HIV burden. Second, YWSS, the target group for this evaluation are a “hidden” population for which a sampling frame does not exist. Third, there are few intervention districts, and 80% of the intended beneficiaries that fall within our target group will be recruited from only two districts. Fourth, there are numerous providers and the strategies to identify, reach and refer YWSS to services is complex.

Our overall approach to these design challenges is to: 1) use existing data (from the *Sisters* programme) to select comparison sites comparable to the DREAMS+PrEP sites in factors known to influence HIV risk; 2) leverage the use of the network-based (respondent driven) strategy that is being used to identify and reach YWSS, refer these YWSS to services, in order to recruit them into the impact evaluation, and repeat this process in the non-DREAMS comparison sites; 3) take a cohort analysis approach to compare HIV incidence among YWSS in the DREAMS+PrEP sites with YWSS in the non-DREAMS comparison sites, and collect data on recent infection and risk factors for HIV incidence at enrolment in support of an adjusted analysis; 4) embed a process evaluation within the impact evaluation to seek to understand intervention implementation, and document any contextual factors determining the success or failure of delivery of intervention components.

## Methods

### Study location and non-randomised site selection

We will conduct this study in two DREAMS+PrEP and four non-DREAMS comparison sites. We selected the two DREAMS+PrEP sites, as these sites will refer the largest proportion of young women to PrEP and supportive interventions. These two sites are cities, among the largest in Zimbabwe, with a combined population of over 500,000. Each site has a static *Sisters* programme clinic that offers walk-in clinical services Monday to Friday each week [9].

We selected comparison sites from the 36 sites where the *Sisters* programme is operational [9]. Of the 36 national *Sisters* sites, six are static sites in larger towns and cities that provide walk-in services on weekdays and 30 are mobile sites in smaller towns where outreach services are delivered weekly. From the *Sisters* sites, we first excluded sites that overlapped with DREAMS, excluded seven sites where an HIV treatment and prevention, including PrEP, intervention trial (SAPPH-Ire trial) had been implemented [17], and a site where another organisation is delivering PrEP. Second, we excluded 15 rural sites. From the remaining eight urban sites, we selected four comparison sites using routine *Sisters* programme data and expert opinion. We selected one site as it is a large town with a static *Sisters* clinic where > 1500 FSW were registered by mid-2016, of whom 25% were aged 18 to 24. Of the remaining seven sites we selected three sites because they each had: 1) > 400 FSW registered with the programme; 2) among whom > 20% were aged 18–24, and 3) each site was a similar distance to Harare as the two DREAMS+PrEP sites.

### Measurements and sample collection

Young women recruited to the impact evaluation study will be asked to provide written consent to complete a questionnaire, provide a dried blood spot (DBS) and have a rapid HIV test. Women will be followed-up at 12 and 24 months, and asked to complete an interviewer-administered questionnaire and offered HIV testing services using a rapid HIV test-kit. The questionnaire will cover socio-demographic factors, a range of behavioural characteristics, access to health and/or social-support services, and HIV-related questions. We will use the DBS collected at enrolment to test for HIV, and, for those who test HIV-positive, to determine prevalence of recent infection using Recent Infection Testing Algorithm (RITA), which includes use of the LAG avidity assay coupled with viral load testing.

### Retention and follow-up procedures

To assist with retention of the cohort, women will be asked to provide their phone number and residential information, and informed that the information will be used to send follow-up reminders via WhatsApp® or through home visits 12- and 24-months after enrolment. We will exclude young women who report that they are planning to move away from the district within the next six months. Locator information will be checked for completeness and accuracy at each visit. To support retention in the programme, curated WhatsApp® broadcast groups of approximately 50 participants each will be developed. The curator will send messages but the broadcasts will not disclose who is in the group or share any phone numbers.

### Primary outcome measurement

The primary outcome is HIV incidence over the 24 months following enrolment into the study, the numerator will be the number of new infections among young women testing HIV-negative at enrolment, the denominator the person-years of follow-up. Person-years of follow-up for each YWSS will be calculated as: 1) time between the first HIV negative test at enrolment and an HIV negative test at 12 months and 24 months; or 2) the mid-point between an HIV negative test and an HIV positive test. If a participant is lost to follow-up (LTFU), the participant will be censored at last HIV-negative test.

### Secondary outcomes

This study has many secondary outcomes, including reduced food insecurity, reduced condom-less sex with sexual partners, reduced experience of violence from partners and police, reduced reliance on sex work for economic reasons, increased uptake of HIV testing services and access to STI treatment services, and self-reported adherence to ART or PrEP.

### Ethical approval

We received ethical approval for this study from the Medical Research Council of Zimbabwe (Reference number MRCZ/A/2085) and the London School of Hygiene and Tropical Medicine (Reference number 11835).

### Statistical analysis

We aim to adhere to the transparent reporting principles of TREND [22]. In addition, we will report on items essential to the network-based (RDS) recruitment as per the STROBE-RDS extension [23]. We will finalise a statistical analysis plan before data collection at 24-months is complete.

### Cohort profile

Using data collected at enrolment, we will describe non-participation among women recruited to the study (ineligible, non-consent, declined to recruit others), the range and mean size of the sample recruited through the network-based (RDS) strategy, and present recruitment trees by site. We will describe unweighted characteristics of study participants and describe the number with missing data for the outcomes as recommended by the STROBE-RDS extension [24]. Although we cannot present non-participation among women not attending study sites, we will describe the mean and range of women who did not recruit peers by site and ask women recruited into the study to estimate how many women they tried to recruit but who refused. We will report any recruitment challenges in line with the STROBE-RDS extension [24].

### RDS diagnostics

Our analysis will account for the network (RDS)-based recruitment strategy. We (JH, FC) previously described our approach to analysis of RDS data and diagnostics to assess whether there is evidence that the sample deviates from the assumptions made by the RDS-2 estimation [21, 25, 26]. Similar diagnostics will be performed for this study using data collected in the questionnaire among all women and a brief follow-up interview among women collecting recruitment incentives to assess whether our sample deviates from RDS-2 assumptions and whether any deviation differs by the intervention and comparison groups, which would have implications for our findings [21]. Using enrolment data, a diagnostics will include: exploring whether the cumulative estimate of our primary and secondary outcomes stabilise over time from “seed” characteristics, and whether estimates from each seed converge [21, 27]. We will assess the assumption that participants are able to accurately report their network size and randomly recruit from within this network using data on network asked size both in the main interview and in the follow-up interview among participants collecting secondary incentives for recruiting peers to the study (although half the women will not receive secondary incentives once sample size). We will conduct a test-retest reliability of this estimate; assessing the proportion of women who, at the point of enrolment, report that a stranger gave them the recruitment voucher.

### Assessing balance across sites

Using enrolment data, we will describe characteristics of the YWSS recruited into the DREAMS+PrEP and non-DREAMS comparison cohorts. To assess whether there is any imbalance across the two cohorts in risk factors for HIV, we will conduct a comparative analysis of the socio-demographic characteristics, sexual behaviours of the YWSS recruited to the impact evaluation, and prevalent and recent infection. Where there is evidence of imbalance in factors likely to influence HIV risk, we will adjust for these individual-level factors in our analysis of the primary outcome as described below.

### Stages of primary outcome analysis

We will adopt a transparent and simple cohort approach to the analysis of the primary outcome. Our approach to inference will consider whether it is plausible that DREAMS+PrEP had an impact on HIV incidence among the target population [28, 29].

We will conduct our primary analysis in three phases. Each stage aims to build the strength of the evidence base of a plausible causal impact of DREAMS on HIV incidence. In phase one, we will take an individual-level cohort analysis approach. At 24-months, we will describe the number of women retained in the cohort and summarise follow-up time (average and total) in the DREAMS+PrEP and comparison sites. We will describe HIV incidence at 24 months in the DREAMS+PrEP and comparison groups with associated 95%CI. We will also describe site-specific estimates of incidence. Using Poisson regression, we will compare the incidence rates at 24-months across the two groups expressed as an unadjusted rate ratio with associated 95%CI presented. To account for the RDS design, we will weight the data by 1/self-reported degree of social network.

The unadjusted rate ratio is subject to confounding by risk factors for HIV that are differential across the two groups in the absence of random allocation and the small number of sites. In phase two, we will estimate a rate ratio adjusted for potential confounders. We will present the adjusted rate ratio with associated 95%CI.

In the third phase, we will assess whether there is evidence for an association between individual-level exposure to DREAMS+PrEP interventions and HIV incidence. For example, we will compare the risk of HIV infection between women self-reporting that they are adherent to PrEP and women who did not take PrEP. We will specify the exact measures of exposure after the year 1 data have been collected, without reference to the outcome data and before the final follow-up.

### Sample size justification

DREAMS aims to reduce HIV incidence among AGYW by 40%. In the absence of a robust estimate of HIV incidence among the target population, we used data from the *Sisters* programme and the MTN-020 Dapivirine vaginal ring trial to determine the required sample size [9, 30]. A cohort analysis of *Sisters* programmatic data estimated that HIV incidence was 10.8% (95%CI: 8.1–16.1) among FSW aged 18–25 years [9]. In the ASPIRE trial, HIV incidence among women aged 18 to 45 was 4.5 per 100 person-years in the placebo arm, 6.2 per 100 person years in the placebo arms in South Africa, 2.6 in Malawi, Uganda and Zimbabwe [30]. Relative to the target population in our study, women in the ASPIRE trial might have been at lower risk of HIV, with only 7% reporting transactional sex in the previous year. Based on these estimates and assuming individual-level analysis, we assume that incidence among YWSS in the absence of DREAMS+PrEP is between 5.0 and 8.0 new infections per 100 person-years. We also anticipate that, at the point of enrolment, 20% of YWSS will test HIV positive and that 30% of YWSS will be LTFU over the 24-month period. Based on these estimates, we will recruit 1200 YWSS in the DREAMS+PrEP sites and 1200 in non-DREAMS sites to have sufficient person-years of follow-up to have 80% power to detect a reduction of 40% or more in incidence after 24-months at the *p* = 0.05 level (Table 1).

**Table 1:** Study power to detect a 30–50% reduction in HIV incidence in DREAMS+PrEP cohort compared to incidence in a comparison cohort with at least 690 YWSS followed up over 24 months (1500–1380 person-years follow-up/cohort)

HIV incidence in comparison cohort	Reduction in HIV incidence in DREAMS+PrEP cohort					
	50%	40%	30%	50%	40%	30%
	1500pyrs follow-up per cohort			1380pyrs follow-up per cohort		
5.0	94	77	49	92	75	46
6.0	96	85	59	96	82	55
7.0	98	90	66	98	87	62
8.0	99	93	72	99	91	67

### Process evaluation

**Fig. 2:**
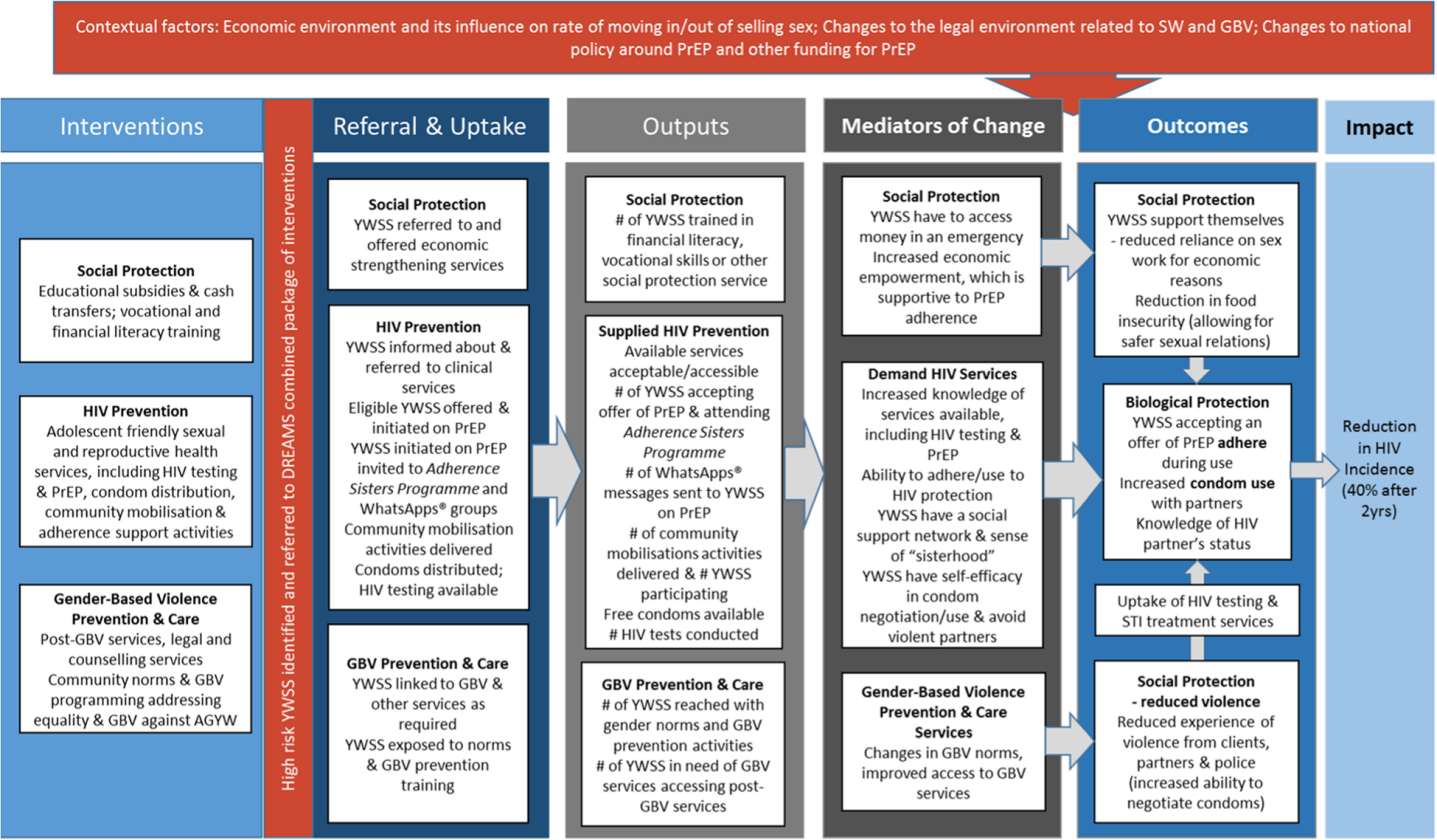
Hypothesised causal pathway of the DREAMS+PrEP package of interventions on HIV incidence

Embedded within the impact evaluation is a process evaluation that will compile routine programme data and conduct qualitative research to assess the fidelity, acceptability, and feasibility of the DREAMS+PrEP package. The process evaluation framework is designed to accompany the assumed pathway of the intervention (Fig. 2). The aim of the process evaluation is to strengthen the plausibility that any apparent effect is attributable to the DREAMS combined package.

### Methods

To determine fidelity, interviews with staff across DREAMS providers will examine whether the programme components were delivered as intended and identify challenges, delays, or omissions in planned implementation. We will also conduct in-depth interviews at the time of the network-based recruitment with five “seeds” in each of the two DREAMS+PrEP and two non-DREAMS sites to discuss issues relevant to the hypothesised causal pathway. In-depth interviews will also be conducted in these four sites with a cross-section of twenty-four (*n* = 24) participants every six-months, purposively sampled according to age and levels of uptake of different DREAMS+PrEP intervention or *Sisters* programme components. Twice a year, participatory evaluation workshops will be conducted with YWSS engaged in DREAMS+PrEP to elucidate their perceptions of the quality and usefulness of different aspects of the programme. Records of outreach contacts, activity attendance and referrals between implementing partners will be analysed to assess the number of DREAMS services that YWSS access, and with what frequency. Finally, a smaller cohort of YWSS will be prospectively followed through repeat interviews and audio diaries (*n* = 12) to explore dimensions of their lives targeted by DREAMS, namely empowerment, financial literacy, personal aspirations, and sexual behaviour and health at individual level, and social support and peer norms at community level.

## Discussion

Estimating the population-level impact of the combined package of interventions delivered through DREAMS on HIV incidence presents numerous challenges, including non-random allocation of DREAMS to a small number of sites, the lack of sampling frame for the target population and high number of service providers. In response, we sought to design a robust study and analysis strategy to allow us to infer whether DREAMS+PrEP has an impact on HIV incidence among YWSS in Zimbabwe. We designed a non-randomised prospective study, that uses a network-based (RDS) recruitment strategy and adopts a cohort analysis approach to compare HIV incidence among cohorts of YWSS recruited in DREAMS+PrEP and non-DREAMS sites.

Nonetheless, our study has limitations. There is no sampling frame for the target population for the evaluation. Our decision to use the same network (RDS)-based strategy attempts to ensure that the populations recruited in the DREAMS+PrEP and non-DREAMS comparison groups are comparable. First proposed in 1997 [31, 32], the use of RDS surveys has become increasingly popular [32]. In parallel, statistical techniques for the analysis of RDS data has evolved to allow for less biased estimation [25]. More recently, studies have used RDS to recruit and follow-up a cohort of individuals, primarily among men who have sex with men [33–36]. Our study team has substantial experience conducting RDS surveys among FSW [10, 11, 21]. Although approximately 30% of FSW recruited to these surveys have been aged 18–24 [10, 11], this study is recruiting a broader population of young women, including those who do not self-identify as FSW.

We recognise that retention of the YWSS recruited into the cohorts may prove challenging, as such our sample size calculations assume a 30% LTFU over the 24-month study. Furthermore, individuals LTFU may differ in the two groups, leading to bias estimates of HIV incidence. We opted for a cohort design rather than repeat cross-sectional surveys as we are interested in the impact of DREAMS+PrEP among YWSS referred to and offered DREAMS+PrEP services. We recognise that both design options have limitations. Repeat cross-sectional surveys, however, would suffer from issues of temporality, we would not be able to explore individual-level changes over time, and women recruited at successive surveys may have had no exposure to DREAMS+PrEP. Through our cohort study design, YWSS will be referred to and offered DREAMS+PrEP services dependent on need. Our study will therefore allow us to understand women’s level of engagement and reasons for non-engagement with DREAMS.

The DREAMS package of interventions aims to reduce HIV incidence by 40% among AGYW. As such, our study is powered to detect a 40% difference in HIV between the DREAMS+PrEP and non-DREAMS comparison cohorts. This is a large reduction in incidence. If DREAMS+PrEP reduces incidence by 40% we may be able to detect this difference, if levels of LTFU and HIV prevalence at enrolment are as hypothesised, but reductions lower than 40% may not be detected with the proposed sample size. PrEP efficacy studies have shown up to 67% reduction in HIV among sero-discordant couples and young men and women in sub-Saharan Africa [1, 37, 38]. Sub-group analyses suggest efficacy is higher with increased levels of adherence [1, 37]. An offer of PrEP alongside interventions to support retention in or return to school, and improve access to HIV testing, STI screening and treatment among other services are likely to have a large impact on these women’s lives, their access to services and their risk of HIV infection. Should this study be underpowered, it can still provide evidence critical to understanding whether and how DREAMS+PrEP changed young women’s lives and to informing how to provide this underserved and vulnerable population with access to HIV prevention and care services within a broader social support package.

Interventions for the prevention of HIV are now available, including PrEP and condoms [18, 39]. Since 2010, there have been declines in HIV incidence many countries, yet young women aged 15–24 years remain at disproportionately high risk, accounting for 20% of new infections [2]. In sub-Saharan Africa, young women accounted for 25% of new adult HIV infections in 2015 [2] Implementation science research is required to identify how best to optimise delivery of these prevention interventions in different settings [18, 40]. Optimal delivery is not straightforward, requiring the implementation of multiple interventions to increase demand, achieve acceptable and accessible supply, and overcome barriers to adherence and use, including socioeconomic barriers [18, 39]. Contextual factors that influence optimal delivery also need to be considered. In order to maximise population impact, implementation research should focus on how to deliver such complex interventions to those AGYW who are at highest risk of HIV infection and hardest to reach with prevention and care programmes [4]. We have described the approach we have taken to evaluate the DREAMS+PrEP intervention among young women at highest risk in Zimbabwe. Transparency with regards to design considerations for evaluating complex prevention programmes in real world settings is critical to maximise our understanding of how best to bring such interventions to scale effectively.

## Abbreviations

AGYW: Adolescent girls and young women
CI: Confidence interval
DREAMS: Determined, Resilient, Empowered, AIDS-free, Mentored and Safe
FSW: Female sex worker
HIV: Human immunodeficiency virus
PrEP: Pre-exposure prophylaxis
RDS: Respondent-driven sampling
YWSS: Young women who sell sex
